# Genetic diversity and population structure of the endangered species *Paeonia decomposita* endemic to China and implications for its conservation

**DOI:** 10.1186/s12870-020-02682-z

**Published:** 2020-11-09

**Authors:** Shi-Quan Wang

**Affiliations:** Ministry of Education Key Laboratory for Ecology of Tropical Islands, Key Laboratory of Tropical Animal and Plant Ecology of Hainan Province, College of Life Sciences, Hainan Normal University, Haikou, 571158 China

**Keywords:** Conservation strategy, Genetic diversity, Genetic relationships, *Paeonia decomposita*, Population structure, Simple sequence repeat (SSR)

## Abstract

**Background:**

*Paeonia decomposita*, endemic to China, has important ornamental, medicinal, and economic value and is regarded as an endangered plant. The genetic diversity and population structure have seldom been described. A conservation management plan is not currently available.

**Results:**

In the present study, 16 pairs of simple sequence repeat (SSR) primers were used to evaluate the genetic diversity and population structure. A total of 122 alleles were obtained with a mean of 7.625 alleles per locus. The expected heterozygosity (*H*_e_) varied from 0.043 to 0.901 (mean 0.492) in 16 primers. Moderate genetic diversity (*H*_e_ = 0.405) among populations was revealed, with Danba identified as the center of genetic diversity. Mantel tests revealed a positive correlation between geographic and genetic distance among populations (*r* = 0.592, *P* = 0.0001), demonstrating consistency with the isolation by distance model. Analysis of molecular variance (AMOVA) indicated that the principal molecular variance existed within populations (73.48%) rather than among populations (26.52%). Bayesian structure analysis and principal coordinate analysis (PCoA) supported the classification of the populations into three clusters.

**Conclusions:**

This is the first study of the genetic diversity and population structure of *P. decomposita* using SSR. Three management units were proposed as conservation measures. The results will be beneficial for the conservation and exploitation of the species, providing a theoretical basis for further research of its evolution and phylogeography.

## Background

The genus *Paeonia* L. (Paeoniaceae) includes 32 woody and herbaceous species, mainly distributed in the northern hemisphere. *Paeonia* is divided into three sections: *Onaepia*, *Moutan*, and *Paeonia* [1, 2]. The *Moutan* section comprises eight species that are native and endemic to China [2] and commonly termed Mudan or tree peonies in Chinese. In China, Mudan is regarded as the ‘King of Flowers’, and the plant is prized both for its pharmaceutical applications and its ornamental value [1, 3]. Seed oil can be extracted from peony seeds, which contain fatty acids, and so the peony has become an important woody oil crop [4].

*Paeonia decomposita* Handel-Mazzetti is a species from the *Moutan* section. It is found principally in the remote mountain areas of northwest Sichuan Province, and is both indigenous and endemic to China, with a sporadic and narrow distribution and small population size. It grows in sparse *Cupressus chengiana* forests, young secondary deciduous broad-leaved forests, and thickets at an altitude of 2000–3100 m and has 2*n* = 10 chromosomes. It is cross-pollinated by insects [5] and propagates by seeds [6]. In the past, *P. decomposita* consisted of two subspecies: *P. decomposita* subsp. *rotundiloba* and *P. decomposita* subsp. *decomposita* [7–9]. Based on morphological traits and molecular data, they are now considered separate species [2, 10, 11].

*P. decomposita* is a celebrated ornamental flower on account of its large, showy, colorful, and fragrant flowers. Thus, local people collect the plants to use in ornamental gardening. It is also a traditional medicinal plant because its root bark (‘Danpi’ in Chinese) is used as a traditional Chinese medicine, having multiple therapeutic properties, for example, clearing heat, cooling blood, activating blood flow, and removing blood stasis [12]. It has recently become considered an important woody oilseed plant. The mean kernel oil content was found to be 32.23 ± 1.96%, consisting of seven fatty acids. Most of the oil (91.94–93.70%) was found to consist of unsaturated fatty acids, with linolenic acid accounting for 40.45–47.68% [13]. The extracted oil from the seeds can be utilized as oleochemicals, cosmetics, and medicines [14]. Therefore, *P. decomposita* is considered to be not only an ornamental plant but also an important officinal plant with a valuable woody oil crop.

Due to multiple threats including habitat damage, excessive harvesting of seeds, misuse of the root-bark in traditional Chinese medicine, and a naturally poor regenerative ability, *P. decomposita*’s natural habitats have become increasingly fragmented, with the natural population size and individual numbers of plants decreasing dramatically, resulting in a significant loss of genetic resources. Currently, most populations are small, fragmented, and scattered, increasing the probability of inbreeding and the potential for genetic drift. Also, low seed production, difficult seedling renewal, and the lack of a specific mechanism for long-distance seed dispersal have resulted in poor population regeneration because many communities are short of seedlings and saplings. Following its distribution, biological characteristics, and survival status, *P. decomposita* has been listed as an endangered plant (EN) [15, 16]. Conservation of the species is therefore critically important. Genetic resource conservation and plant breeding programs require an evaluation of the genetic diversity and structure of the endangered species [17, 18]. However, it is difficult to plan conservation strategies for this plant due to a lack of genetic background knowledge.

The use of molecular markers allows precise estimates of genetic diversity. In the past, researchers have used various molecular markers, including amplified fragment length polymorphisms (AFLP), sequence-related amplified polymorphisms (SRAP), inter simple sequence repeats (ISSR), and random amplified polymorphic DNAs (RAPD), to study the genetic relationships among the species in Section *Moutan* [19–23]. Compared with AFLP, SRAP, ISSR, and RAPD, simple sequence repeats (SSR) markers have the significant advantages of co-dominance, wide distribution, high transferability, high polymorphism, high reproducibility, and high reliability combined with relatively low expense [24, 25], resulting in them being commonly regarded as ideal molecular markers. They have been widely employed to study genetic diversity, population structure, and the genetic relationships of different plant species [26–30], including tree peonies [31–33].

To date, the study of *P. decomposita* has been limited to the genetic relationships among species and the genetic diversity of ISSRs [34], with no studies exploring the genetic diversity of SSRs, or the genetic relationships or population structures of this important woody oilseed species. No breeding plan has been established from which to select an optimum germplasm or resource conservation strategy, hindering the conservation of *P. decomposita*. Thus, an accurate understanding of the population structure and genetic diversity of *P. decomposita* is urgently required.

Accordingly, given its value in medical, industrial, and ornamental applications, a genetic study of the plant was conducted. In the present study, I first selected 16 pairs of polymorphic SSR markers, then evaluated the molecular variance among and within populations to determine the genetic diversity and population structure, to provide crucial information for establishing an appropriate conservation and management strategy of genetic germplasm resources and the deployment of these resources with plans for a future directive breeding strategy.

## Results

### SSR marker polymorphism

In the present study, a total of 122 alleles at 16 polymorphic microsatellite loci [35–43] (Table 1) were amplified across 258 individual plants from 11 natural populations. The number of observed alleles per locus (*N*_a_) varied greatly among loci, from two alleles (locus PSMP2) to 20 alleles (locus PAG1) (mean = 7.625). The number of effective alleles per locus (*N*_e_) ranged from 1.045 (locus PSMP2) to 9.929 (locus PAG1) (mean = 3.208). The observed heterozygosity per locus (*H*_o_) ranged from 0.027 (locus WD09) to 0.992 (locus 73A) (mean = 0.385), whereas the expected heterozygosity (*H*_e_) ranged from 0.043 (locus PSMP2) to 0.901(locus PAG1), with a mean of 0.492. The polymorphic information content (*PIC* value) of the primers varied from 0.042 (locus PSMP2) to 0.891 (locus PAG1) with a mean of 0.456. In total, 21 private alleles (*N*_P_) were identified in 9 populations except for M2 and M4 by 16 markers (Table S1).

**Table 1:** Characteristics of 16 polymorphic microsatellite primers

Locus	Primer sequence (5′–3′)	Repeat motif	Reference
50F, R	F: AGAAGAGTAACATGCGCC	(CT)_10_	[35]
	R: AAGACCTCCACTGCAGAT		
56A	F: CAGGTGGCATTTTTGGCTTCTCTCT	(AC)_15_	[36]
	R: TTGGCCCAATCACATGTAATCCCTC		
73A	F: CCATCTCAGGGTCAGGGTTCTCGTA	(CAG)_5_	[36]
	R: TAGAGTGTACCTTCACCCCCATCGG		
91A	F: TCAGCCCCTAGCATAGAAGAATCCA	(GT)_9_TTGTA(TG)_16_	[36]
	R: TCTCACTACCACCTACGCGATGTTC		
PAG1	F: AGTGGTGGAAGATTGGAC	(AG)_24_	[37]
	R: AAATACTCCGTCTTAGTGTGAA		
AG8073	F: TCAGCTAATATGGGTGTTTC	(AG)_10_	[37]
	R: ATCAAAGTGGAAGTTCTACAGT		
P03	F: ATGTCACCGAAAGTTGTGC	(GA)_10_	[38]
	R: AAAGCCTGGTGCAGTTATT		
P05	F: TCGCCCAACCTGTCGTGGAGAT	(AG)_9_	[38]
	R: TTGAATAGAGCGGAATGGAAAA		
P10	F: CACAAAACTCCTTCATCTTC	(CT)_20_	[38]
	R: ATCGTCAATTAGAATCAGAC		
P12	F: TTGGTTGGTGAAGGTGTT	(TC)_9_TTTCTCTCTA(TC)_5_	[38]
	R: CTTCGATAACCGCAGGAGGAT		
PSESP5	F: GCTCATTACCGCTACTACCA	(A)_26_	[39]
	R: AAAACCACTCACCTCCCA		
PSMP2	F: GACTATTTTGCCCCAGACAT	(ATTT)_7_	[40]
	R: AAGATACAAGCAGTTCACGC		
WD09	F: GGGGACTCAAATCCTTGCGAAAACCA	(CAC)_4_	[41]
	R: AGGCCTAGTTTTGGTCTGGGCG		
Pae100	F: ACCATTCAAGGTGAGCTTCC	(AT)_7_	[42]
	R: TCCAGATATATTCCCTCACCCTA		
PS004	F: GTGCTTAGCCTCTAATCTG	(GA)_8_	[43]
	R: CTTTGCTCCAAGTCTGTC		
PS026	F: TTCCCTCCATTCTAACAC	(AG)_6_	[43]
	R: ACCCTAGCCTCTGACATT		

At the locus level, the genetic differentiation coefficient (*F*_st_) and gene flow (*N*_m_) calculated from *F*-statistics at each locus in the species were significantly different. The paired comparison of genetic differentiation between the populations indicated that the maximum values of *F*_st_ and *N*_m_ were 0.430 (at locus 50F, R) and 2.735 (at locus PSESP5), respectively. The genetic differentiation coefficient (*F*_st_) was estimated to be 0.193 for the 16 loci (ranging from 0.084 at locus PSESP5 to 0.430 at 50F, R). The mean inbreeding coefficient (*F*_is_) was 0.038 (Table 2).

**Table 2:** Statistical values of microsatellite markers on 258 samples across 11 populations of *Paeonia decomposita* in China

Locus	*N*_a_	*N*_e_	*I*	*H*_o_	*H*_e_	*F*	*PIC*	*A*_r_	*F*_is_	*F*_st_	*N*_m_
50F, R	3	1.293	0.440	0.125	0.227	0.448	0.209	3	− 0.026	0.430	0.331
56A	15	5.355	1.926	0.395	0.815	0.515	0.789	14.552	0.374	0.195	1.033
73A	3	2.548	1.003	0.992	0.609	−0.632	0.530	3	−0.784	0.091	2.502
91A	13	7.624	2.214	0.553	0.871	0.363	0.856	12.981	0.308	0.138	1.556
PAG1	20	9.929	2.540	0.590	0.901	0.344	0.891	19.972	0.154	0.153	1.385
AG8073	3	1.252	0.408	0.113	0.202	0.440	0.189	3	0.148	0.302	0.577
P03	3	1.803	0.690	0.401	0.446	0.101	0.359	3	−0.182	0.255	0.732
P05	5	2.145	0.851	0.416	0.535	0.220	0.425	4.848	0.081	0.175	1.175
P10	7	2.443	1.183	0.486	0.592	0.177	0.547	6.883	−0.001	0.146	1.467
P12	15	5.388	1.977	0.711	0.816	0.127	0.793	14.745	−0.035	0.139	1.552
PSESP5	4	1.122	0.272	0.093	0.109	0.132	0.106	4	0.062	0.084	2.735
PSMP2	2	1.045	0.106	0.036	0.043	0.160	0.042	2	−0.002	0.151	1.400
WD09	3	1.049	0.128	0.027	0.046	0.418	0.046	3	0.245	0.211	0.936
Pae100	8	1.484	0.762	0.187	0.327	0.427	0.313	7.886	0.284	0.235	0.815
PS004	12	4.682	1.848	0.687	0.788	0.127	0.762	11.865	−0.031	0.139	1.554
PS026	6	2.161	0.868	0.345	0.538	0.357	0.431	5.689	0.020	0.250	0.751
mean	7.625	3.208	1.076	0.385	0.492	0.233	0.456	7.526	0.038	0.193	1.281

*N*_a_: The observed number of allele, *N*_e_: The effective number of alleles, *I*: Shannon’s information index, *H*_o_: Observed heterozygosity, *H*_e_: Expected heterozygosity, *F*: Fixation index, *PIC*: Polymorphism information content, *A*_r_: Allelic richness, *F*_is_: Inbreeding coefficient among individuals within populations, *F*_st_: Average genetic differentiation coefficienct, *N*_m_: Gene flow

### Population genetic diversity

At the population level, genetic diversity indices (in terms of *PPL*, *N*_a_, *N*_e_, *I*, *H*_o_, *H*_e_, *F*) varied across populations of *P. decomposita*, as listed in Table 3. On average, the percentage of polymorphic loci (*PPL*) across eleven populations was high (80.68%) and ranged from 68.75% for JC5 to 93.75% for DB1, with most populations (10/11) ≥ 75%. The number of observed alleles (*N*_a_) per population varied from 2.563 (M4) to 4.813 (DB2), with a mean of 3.637. The number of effective alleles (*N*_e_) across all populations was 2.322, varying from 1.811 (M1) to 2.813 (DB2). The mean heterozygosity (*H*_e_) and observed heterozygosity (*H*_o_) across all populations ranged from 0.329 (M1) to 0.538 (DB1) and 0.314 (M2) to 0.464 (DB2), with means of 0.405 and 0.394, respectively. The mean value of Shannon’s Information Index (*I*) was 0.777 over a range of 0.580 (M1) to 1.017 (DB1). The fixation index (*F*) averaged 0.032, ranging from − 0.160 (JC5) to 0.154 (DB1) at the population level. The majority of loci were in accord with the Hardy–Weinberg Equilibrium (HWE), but several populations did not fully satisfy the HWE, especially populations DB2 and M3 in which many loci were found to deviate from the HWE (7 and 8 loci, respectively), indicating a panmictic population structure. Loci 56A and 73A deviated from HWE in all populations.

**Table 3:** Genetic variation of the 11 populations in *Paeonia decomposita*

Pop	*N*_a_	*N*_e_	*I*	*H*_o_	*H*_e_	*F*	*PIC*	*A*_*r*_	*PPL*	*Fis*	*HWE*
DB1	4.313	2.714	1.017	0.456	0.538	0.154	0.456	3.731	93.75%	0.178	56A^*^, 73A^***^, 91A^***^, PAG1^*^, PSESP5^***^, PS004^**^
DB2	4.813	2.813	0.989	0.464	0.486	0.053	0.464	3.892	87.50%	0.066	56A^**^, 73A^***^, 91A^*^, AG8073^*^, P05^***^, PAG1^***^, Pae100^*^
JC1	3.875	2.616	0.826	0.437	0.407	−0.045	0.437	3.666	75.00%	−0.032	56A^*^, 73A^**^, 91A^***^, PS026^**^
JC2	3.500	2.002	0.711	0.358	0.374	0.101	0.358	2.959	81.25%	0.063	56A^**^, 73A^***^, 91A^*^, AG8073^**^, Pae100^**^
JC3	4.250	2.779	0.934	0.408	0.454	0.055	0.408	3.802	87.50%	0.127	56A^***^, 73A^***^, 91A^***^, P05^**^, Pae100^***^
JC4	3.438	1.924	0.733	0.425	0.399	−0.022	0.425	2.953	87.50%	−0.040	56A^***^, 73A^***^, P10^*^, Pae100^***^
JC5	3.063	2.132	0.707	0.438	0.388	−0.160	0.438	2.779	68.75%	−0.098	56A^*^, 73A^***^, PAG1^*^
M1	3.063	1.811	0.580	0.320	0.329	0.000	0.320	2.365	81.25%	0.041	56A^***^, 73A^***^, 91A^**^, PAG1^***^, PS004^***^
M2	3.625	2.307	0.689	0.314	0.352	0.065	0.314	2.869	75.00%	0.120	56A^***^, 73A^***^, 91A^*^, PAG1^***^, PS004^***^, PS026^*^
M3	3.500	2.584	0.767	0.385	0.398	0.079	0.385	3.087	75.00%	0.055	56A^***^, 73A^***^, P03^**^, P05^*^, P10^*^, Pae100^***^, PS004^**^, PS026^*^
M4	2.563	1.859	0.593	0.325	0.335	0.071	0.325	2.563	75.00%	0.083	56A^*^, 73A^**^, Pae100^**^
mean	3.637	2.322	0.777	0.394	0.405	0.032	0.394	3.151	80.68%	0.051	

*N*_a_: The observed number of allele, *N*_e_: The effective number of alleles, *I*: Shannon’s information index, *H*_o_: Observed heterozygosity, *H*_e_: Expected heterozygosity, *F*: Fixation index, *PIC*: Polymorphism information content

*A*_r_: Allelic richness, *PPL*: the percentage of polymorphic loci, *F*_is_: Inbreeding coefficient among individuals within populations, HWE: loci showing a significant departure from Hardy-Weinberg equilibrium with a global test at 5% level and after a sequential Bonferroni correction

(*P < 0.05. ** *P* < 0.01. *** *P* < 0.001. indicates loci with heterozygote deficit)

### Genetic differentiation and gene flow between populations

The difference in genetic differentiation (*F*_st_) between pairs of populations was highly significant (*P* < 0.001), varying from 0.041 (between JC1 and JC2) to 0.234 (between DB2 and M2), with a mean value of 0.098 (*P* < 0.001; Table 4), measured across 11 populations based on 16 markers. Conversely, the values for gene flow (*N*_m_) between populations varied from 0.820 (between DB2 and M2) to 5.890 (between JC1 and JC2), with a mean value of 2.781 (Table 4).

**Table 4:** Genetic differentiation coefficient F_st_ (below diagonal) and gene flow *N*_m_ (above diagonal) between populations

	DB1	DB2	JC1	JC2	JC3	JC4	JC5	M1	M2	M3	M4
DB1	–	3.606	2.404	2.310	2.380	2.213	2.204	2.238	1.588	2.352	1.609
DB2	0.065	–	1.372	1.143	1.304	1.264	0.947	1.313	**0.820**	1.267	0.887
JC1	0.094	0.154	–	**5.890**	3.710	3.981	3.238	3.153	3.033	4.420	2.941
JC2	0.098	0.179	**0.041**	–	4.516	4.183	3.726	3.319	2.818	3.640	3.255
JC3	0.095	0.161	0.063	0.052	–	2.814	2.223	2.752	2.861	3.964	3.443
JC4	0.102	0.165	0.059	0.056	0.082	–	2.893	2.107	2.139	2.220	2.195
JC5	0.102	0.209	0.072	0.063	0.101	0.080	–	2.410	2.577	2.270	2.296
M1	0.100	0.160	0.073	0.070	0.083	0.106	0.094	–	2.043	3.816	2.691
M2	0.136	**0.234**	0.076	0.081	0.080	0.105	0.088	0.109	–	5.108	5.493
M3	0.096	0.165	0.054	0.064	0.059	0.101	0.099	0.061	0.047	–	5.592
M4	0.134	0.220	0.078	0.071	0.068	0.102	0.098	0.085	0.044	0.043	–

**Fig. 1:**
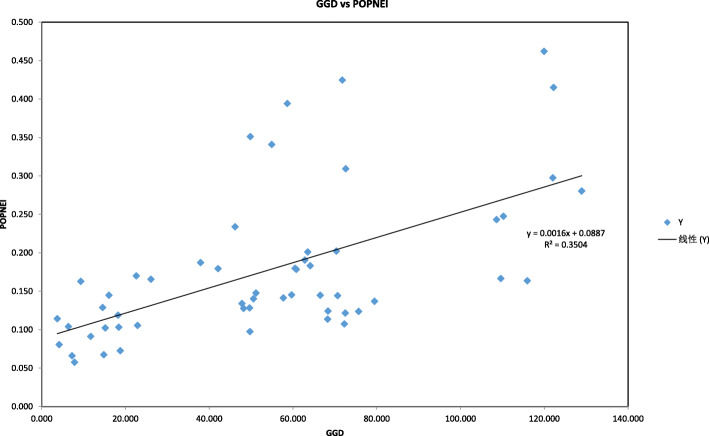
Correlation test of genetic distance (GD) and geographic distance (GGD)

**Fig. 2:**
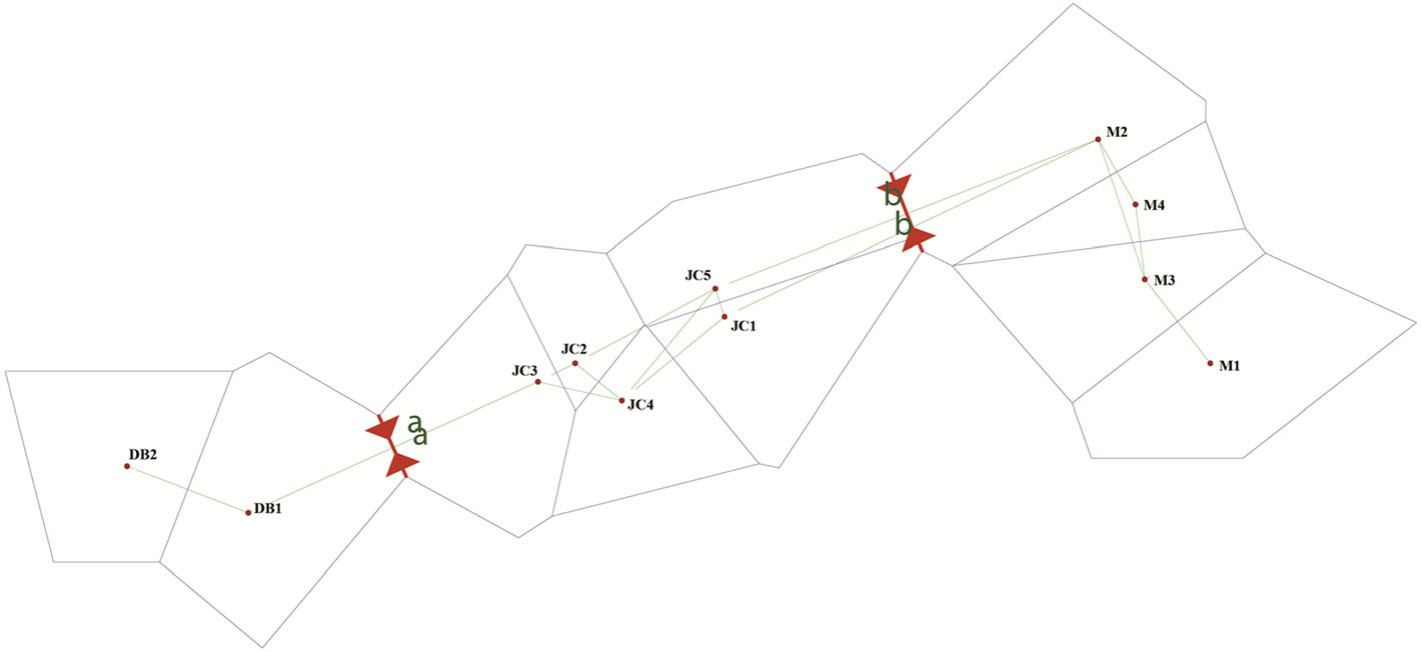
Barriers to the flow of genes. Gray lines correspond to hypothetical boundaries between populations, which are labeled with corresponding codes. Red solid lines with arrows are used to indicate barriers to the flow of genes, and population abbreviations are as represented for Table 7

Nei’s genetic distance, calculated from a pairwise comparison, varied from 0.058 (between M2 and M4) to 0.462 (between DB2 and M2) based on SSR markers, with a mean value of 0.178, and the majority of pairwise genetic distances occurring over the range 0.1–0.3 (Table 5). A Mantel test conducted for *P. decomposita* indicated a positive correlation between geographic and genetic distance among populations (*r* = 0.592, *P* < 0.001) (Fig. 1), in line with the IBD (isolation by distance) model. Results of the AMOVA demonstrated that 81.70% of the total molecular variance was due to differences within regions, while the remainder (18.30%) occurred among regions (*P* < 0.001). At the population level, 73.48% of total molecular variance resulted predominantly from individual differentiation within populations, the remainder (only 26.52%) resulting from molecular variance among populations (all *P* < 0.001). When total molecular variance was grouped into three hierarchical components, analysis by AMOVA revealed that the proportion of maximum molecular variance (70.61%) was still brought about by genetic differentiation within populations (*P* < 0.001), whereas 13.39% (*P* < 0.001) and 16% (*P* < 0.001) of the total molecular variance resulted from genetic differentiation among regions and populations within regions, respectively (Table 6). The positions of inferred gene flow barriers between regions were identified based upon the matrix of *F*_st_ values (Fig. 2).

**Table 5:** Nei’s genetic distances (below diagonal) and Nei’s genetic identity values (above diagonal) are given below for 11 populations. Bold character indicates the highest value, while italic bold character displays the lowest value

Neiʼs Genetic Distance vs Neiʼs Genetic Identity	DB1	DB2	JC1	JC2	JC3	JC4	JC5	M1	M2	M3	M4
DB1	–	0.879	0.837	0.836	0.829	0.792	0.835	0.849	0.784	0.847	0.781
DB2	0.129	–	0.734	0.711	0.704	0.674	0.654	0.756	***0.630***	0.743	0.660
JC1	0.178	0.309	–	0.930	0.900	0.903	0.892	0.868	0.875	0.907	0.880
JC2	0.179	0.341	0.073	–	0.923	0.901	0.902	0.884	0.865	0.893	0.883
JC3	0.187	0.351	0.105	0.081	–	0.850	0.844	0.872	0.866	0.898	0.885
JC4	0.234	0.394	0.102	0.104	0.163	–	0.865	0.817	0.827	0.818	0.833
JC5	0.180	0.425	0.114	0.103	0.170	0.145	–	0.865	0.880	0.863	0.869
M1	0.164	0.280	0.141	0.124	0.137	0.202	0.145	–	0.847	0.913	0.888
M2	0.243	**0.462**	0.134	0.145	0.144	0.190	0.128	0.166	–	0.935	**0.944**
M3	0.167	0.298	0.097	0.114	0.107	0.201	0.148	0.091	0.067	–	0.936
M4	0.247	0.415	0.128	0.124	0.122	0.183	0.140	0.119	***0.058***	0.066	–

**Table 6:** Analysis of molecular variance (AMOVA) for 11 populations of *Paeonia decomposita*

Source of variance	Degree of freedom	Sum of squares	Variance components	Total variance(%)	*P*-value
Among regions	2	348.45	2.02	18.30	< 0.001
Within regions	255	2299.61	9.02	81.70	< 0.001
Among populations	10	726.45	2.81	26.52	< 0.001
Within populations	247	1921.60	7.78	73.48	< 0.001
Among regions	2	348.45	1.47	13.39	< 0.001
Among populations within regions	8	378.01	1.76	16.00	< 0.001
Within populations	247	1921.60	7.78	70.61	< 0.001

### Population structure and genetic relationships

**Fig. 3:**
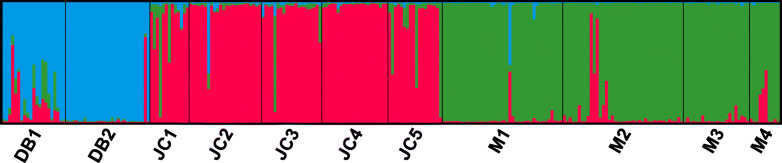
Genetic structure of 11 populations as inferred by STRUCTURE with SSR markers data set

**Fig. 4:**
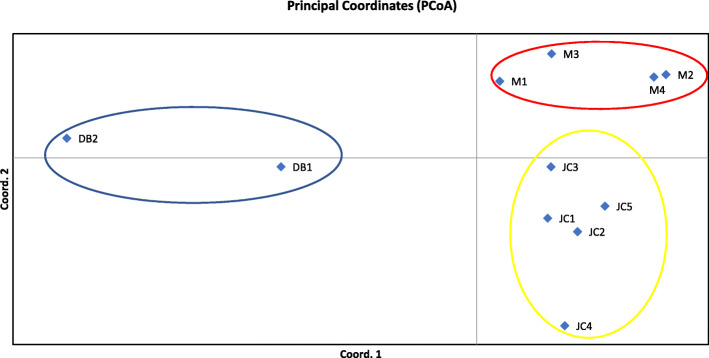
Principal Coordinate Analysis (PCoA) plot of the 11 populations showing three main clusters

The optimal number of genetic clusters equaled 3 when Δ*K* was at its maximum for *K* = 3 (Fig. S1). Thus, all 11 populations under study were split into three distinct genetic clusters (Fig. 3). Cluster 1 contained 49 individual plants collected from two populations in Danba county, Cluster 2 consisted of 97 individual plants sampled from five populations in Jinchuan county, and the remaining 112 arising from four populations in Maerkang county were assigned to Cluster 3. It was apparent that the three genetic clusters were identical to the clusters identified in PcoA, representing the natural distribution of *P. decomposita*. Principal coordinate analysis (PCoA) obtained according to the genetic distance between populations revealed a genetic structure that is presented in Fig. 4. The percentage variance attributable to the three principal coordinate axes was 76.66% (axis 1–50.71%, axis 2–16.95%, and axis 3–9.00%). Furthermore, the results of the PCoA were consistent with those of the structure analysis and supported the UPGMA clustered tree, as described below.

**Fig. 5:**
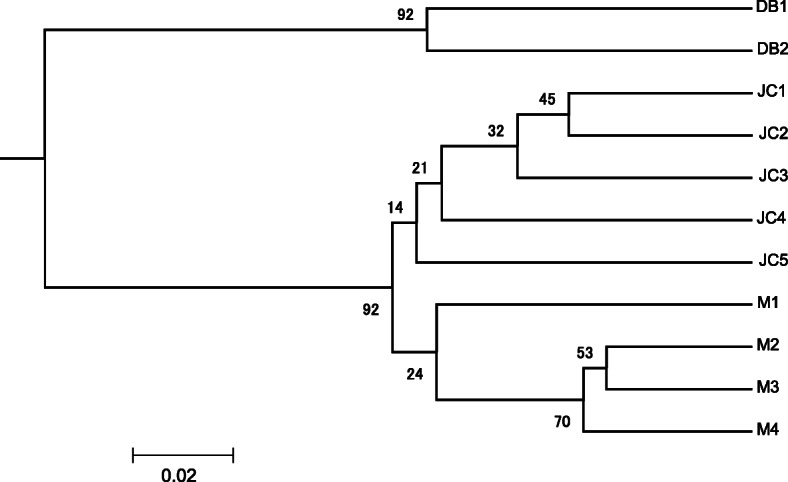
UPGMA dendrogram based on Nei’ genetic distance using SSR marker analysis. The branch length represents genetic distance and the value on the branch is the support rate

The UPGMA dendrogram was constructed from Nei’s genetic distance values and is an accurate reflection of the genetic relationships among and within populations. The UPGMA tree indicated that the 11 populations could be divided into two major clusters: 1 and 2 (Fig. 5). Cluster 1 included two populations, namely DB1 and DB2, with cluster 2 consisting of the remaining 9 populations, which were further divided into two short branches: five populations (JC1, JC2, JC3, JC4, and JC5) from Jinchuan county formed one short branch and four (M1, M2, M3, and M4) from Maerkang county formed another.

## Discussion

It is important to maintain the genetic diversity of natural populations to ensure the continued survival, fitness, and evolutionary potential of a species [44]. Traditionally, the analysis of differences in plant morphology and physiological traits have been used to evaluate diversity. However, only limited information was available for this species using these methods because such traits are not stable under different environmental conditions. Recently, a range of DNA molecular marker techniques have been used to analyse tree peonies, including the use of RFLP [21], RAPD [45], ISSR [46], and AFLP markers [47]. However, these studies were focused on investigating the phylogenetic relationships among interspecies or wild species and it is generally recognized that a greater number of molecular markers are required to conduct genetic studies of *Paeonia* species. SSR is the most practical molecular marker in studies of population genetics because it can measure codominant alleles and display high levels of polymorphism. The present study is the first to investigate the genetic diversity and population structure of *P. decomposita* through microsatellite markers, important for the conservation, management, and greater understanding of its genetic relationships.

### Genetic diversity

Differences in genetic diversity may result from a small number of factors, for example, the life-history or geographic traits of a species [48]. In general, less genetic diversity exists in an endemic species that is not widely distributed compared with that found in a widespread species [49], usually because their population numbers are limited, and as they are isolated from other populations they adapt to their particular habitat [50].

This study demonstrated that the genetic diversity level of *P. decomposita* was moderate (*H*_o_ = 0.394, *H*_e_ = 0.405) among *Paeonia* species even though it is a rare and endangered species. Compared with previous research of wild tree peonies, the genetic diversity parameters observed in this study were slightly lower than those of *P. jishanensis* (*H*_o_ = 0.446) [31] and *P. rockii* (*H*_o_ = 0.459, *H*_e_ = 0.492) [33], but higher than those of *P. ostii* (*H*_o_ = 0.343, *H*_e_ = 0.321) [51], *P. jishanensis* (*H*_e_ = 0.340) [31], *P. delavayi* (*H*_o_ = 0.334, *H*_e_ = 0.369) [52] and *P. ludlowii* (*H*_o_ = 0.014, *H*_e_ = 0.013) [52]. Genetic diversity analysis using ISSR markers indicated a level for *P. decomposita* that was not high [34] and lower than the results of this study.

Levels of genetic diversity in *P. decomposita* (*H*_e_ = 0.405) were lower than both “endemic” species (*H*_e_ = 0.420) and “widespread” species (*H*_e_ = 0.620) [48]. The possible reason for this result is that the sporadic and narrow distribution range, as well as the small sizes of populations and large spatial distances between populations limit pollination among populations, resulting in selfing and inbreeding and potentially leading to low genetic diversity.

The current methods of analysis have considerably improved the understanding of genetic diversity in populations of *P. decomposita*, in which the polymorphism levels varied between populations. In this study, genetic diversity (*I*, *H*_o_, *H*_e_, *PIC*) at the population level was relatively uniform and relatively higher in populations DB1 and DB2 than in other populations, related to low levels of human disturbance and a large population size in Danba. Therefore, Danba represents the major genetic diversity center of the species. Estimation of the fixation index (*F*) revealed that three populations (JC1, JC4, JC5: negative values) displayed an excess of heterozygotes, indicating outbreeding while the other eight populations (positive value) had an excess of homozygotes associated with inbreeding. The mean positive inbreeding coefficient (*F*_is_) values (0.051) indicated an excess of homozygotes in *P. decomposita* (Table 3).

The results strengthened the assumption that endangered plants within a narrow distribution are generally aplastic. A reduction in genetic variation might suggest a decline in adaptation to a changing environment, leading to an increased danger of extinction and increased inbreeding [44, 53].

### Gene flow and genetic differentiation

Two important parameters, gene flow and the genetic differentiation coefficient, are employed to assess the genetic structure of a population [54]. Gene flow and the genetic differentiation coefficient are negatively correlated [55].

Gene flow is a basic micro-evolutionary phenomenon that prevents genetic differentiation among populations and affects the maintenance of genetic diversity [56, 57]. Many endangered plants are isolated and narrowly distributed within a few small populations, possibly leftovers of a formerly widespread species that had a large and continuous population [56, 58]. In the present study, the gene flow (mean *N*_m_ value) between *P. decomposita* populations was > 1, which, in theory, prevents genetic differentiation resulting from genetic drift [59]. Genetic drift has not yet become a predominant factor influencing the genetic structure of *P. decomposita*. However, *P. decomposita* populations are now affected by fragmentation and vandalism, with genetic exchanges occurring within most populations. For these reasons, together with the fact that natural populations are spatially distant (isolated by mountain and river barriers), genetic drift may occur gradually.

Although diversity appears to have occurred mostly within populations, the majority of the genetic differentiation between populations has occurred at a moderate and low level except for a high level of genetic differentiation between DB2 and populations from Jinchuan and Maerkang (Table 4), according to the scale suggested by Wright [60]. The mean *F*_st_ indicates moderate genetic differentiation among the populations of the species. Barrier 2.2 was used to identify barriers to dispersal, revealing that gene exchange was inhibited by the complex terrains among different geographic regions.

The AMOVA results (*P* < 0.001) also support population differentiation. AMOVA revealed the presence of molecular variance among and within populations, with major molecular variance within populations rather than among populations, a situation identical to that observed with other cross-pollinating species in *Paeonia* [33, 51, 52] and other studies using ISSR markers [34]. In outcrossing and long-lived plants in general, most of their genetic variation exists within populations, while selfing plants maintain the majority of genetic variation among populations [48].

### Population structure and genetic relationships

A variety of methods are used to detect genetic diversity and population structure [61–65]. It is advisable to combine three effective techniques and so I consider that the combination of PCoA, Structure, and UPGMA analysis is able to produce reliable results. UPGMA was able to expound intuitive relationships although it cannot fully categorize populations. Conversely, Structure software can objectively categorize populations and produce plans for breeding. Therefore, this method was regarded as the most suitable to categorize populations.

In the present study, UPGMA cluster analysis grouped 11 populations collected from three different regions into two clusters, demonstrating that there were two distinct genetic groups in these areas. The results of Structure clearly suggest that the sampling locations behave as three clusters, with some examples of admixed individuals. These signs of admixture suggest that gene flow may still exist among some locations (which is corroborated by the greater *N*_m_ estimates for some pairs of populations). This suggests that analyses by Structure software were reliable. Furthermore, the PCoA results were identical to those from Structure and supported the UPGMA clustered tree.

In addition, the genetic relationships among populations reflected those populations’ natural geographical locations which were supported by an IBD (isolation-by-distance) model constructed using a Mantel test. This IBD model for *P. decomposita* indicated a positive correlation (*r* = 0.592, *P* < 0.001) between geographic distance and genetic distance between populations. The differences in genetic differentiation were due to geographic barriers, which isolated different gene pools. Inefficient pollen flow, close seed dispersal, and low germination rates are latent reasons which have led to three distinct *P. decomposita* gene pools.

### Conservation of populations in situ and ex situ

It is essential to understand the genetic diversity, structure, and gene flow of a population to create an appropriate management and conservation strategy. The population resources employed for reintroduction, including reproduction material and germplasm collection must be optimal in terms of genetic variation.

The management of collections and conservation of genetic resources must guarantee that most of the existing variation is conserved. Conservation of diversity among populations must concentrate on maintaining the most genetically distinctive populations while conservation of diversity within populations must conserve large core populations in which diversity is not lost due to genetic drift [66]. In the case of *P. decomposita*, conservation must consider not only the geographic distance between the populations, but also the existence of different clusters and their different growth habitats. In every cluster, the priorities for the conservation of populations must be selected, by considering the level of genetic diversity, the state of populations’ regeneration, and their level of threat. Construction of large reserves with several populations in every cluster could guarantee a sample of the gene pool, which could embrace the uniqueness and diversity that exists in all populations.

Genetic diversity is especially important for a species in preserving the latent evolutionary capacity to deal with changing environments [67]. The maintenance of genetic diversity and evolutionary potential is a primary goal for the conservation of endangered species in management programs [68, 69]. Therefore, information about genetic variation within and among populations in endangered and rare plants plays an important role in the process of formulating conservation and management strategies [70]. Thus, I suggest that the three natural distribution areas should correspond to three conservation management units. In view of the current circumstances in which a rapid fall in the number of populations and the extreme endangerment of their natural habitats, in situ and ex situ conservation actions are imperative. All populations, particularly those with high levels of genetic diversity or those with large genetic differences, should be protected. In situ conservation is considered the most effective method of protecting endangered plants, through which the whole gene pool can be protected in a natural habitat. Small populations are more likely to become extinct due to habitat damage and environmental fluctuation. It is essential to conserve all individual plants and populations in situ for the sake of preserving genetic variation as far as possible. Traditional methods of protection that primarily concentrate on in situ conservation, such as improving regeneration, controlling overgrazing, and protecting natural habitats, may be sufficient to maintain the size of the population. Consequently, it is essential to prevent the populations’ genetic homogeneity. In situ conservation must be introduced promptly by defining and introducing conservation reserves in core distribution regions and strictly prohibiting the harvesting of wild *P. decomposita*. Populations DB1 and DB2, with relatively higher genetic diversity than other populations, must be given priority for conservation in situ. Much previous research has demonstrated that heterozygosity is the best method of ensuring populations’ fitness and potential for adaptation [71]. However, a notable heterozygote deficit was found to exist in some populations, including DB1, JC3, and M2, possibly a result of inbreeding in fragments of populations.

The populations of *P. decomposita* are facing the problems of habitat destruction, loss or fragmentation as a result of grazing (M2, JC5, DB1, DB2), over-harvesting (M2, M3), abusive seed collection (JC2–5), growing close to villages, farm fields, and orchards (JC2–5), or areas practically destroyed by urban expansion (M2, M4). Given this challenge, in addition to in situ conservation, it is very much advised that gene banks in both the field and laboratory are established ex situ for each population for which protection is required for endangered plants [72]. The conservation strategy for *P. decomposita* should be aimed at preserving the three detected genetic clusters and taking into account the populations with private alleles (except M2 and M4), for taking conservation actions. Populations DB1 and DB2, with relatively higher genetic diversity than the other populations, must be concrete goals for ex situ conservation. Because the degree of genetic differentiation was low among populations, each may represent a large component of genetic variation in a species. Thus, seed collection tactics could be devised for the construction of an ex situ seed germplasm resource bank to collect as many samples of each population as possible from the whole natural geographical distribution with different genetic clusters, and conserve the germplasm using plant tissue culture techniques. In the course of ex situ conservation, artificial hybridization must be performed among populations with large genetic differences to rapidly improve heterozygosity. After ex situ cultivation of seeds collected from the field, saplings should be introduced into source sites. To summarize, in situ and ex situ conservation methods should be combined to protect valuable genetic resources.

## Conclusions

Genetic information from this detailed study has provided first-hand data of the genetic diversity and population structure of *P. decomposita*, which are beneficial for developing measures to conserve and manage endangered plants. Natural populations maintained moderate to low genetic diversity levels, high gene flow, and low genetic differentiation among populations. Eleven natural populations were categorized into three groups/clusters, which should possibly be considered as three management units for the objective of conservation. These populations are precious genetic resources for a future breeding plan and conservation strategy. This is the first time that the genetic diversity of *P. decomposita* has been studied using SSR, the results representing a reference for improving the germplasm and parental selection for breeding strategy plans.

The markers used in the present study allowed investigation of population structure, genetic diversity, germplasm collection, and conservation strategy for *P. decomposita*. Important information about the genetic structure was provided by these markers, which significantly contribute to future improvements and breeding plans for the species. The genetic diversity, population structure, and genetic relationships between populations through SSR analysis will be helpful for crop breeding, germplasm management, and conservation. To conclude, these results provide value as important resources to study genetic diversity, assist conservation, management, and research plans in the future.

## Methods

### Plant materials

**Fig. 6:**
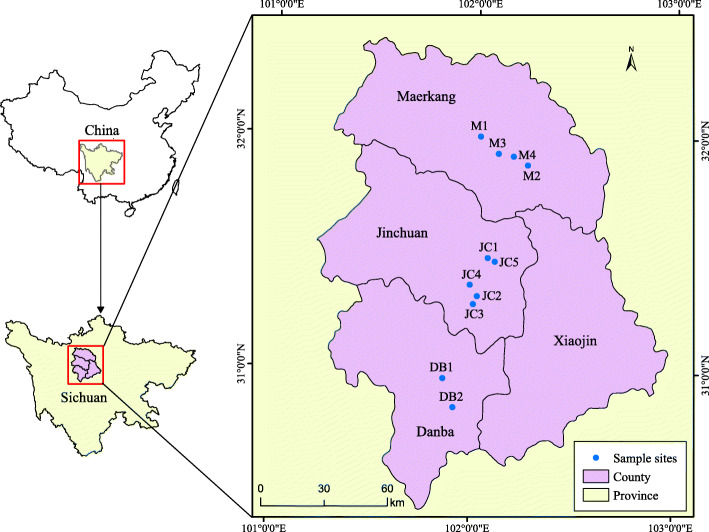
Geographic locations of *P. decomposita* populations sampled in this study

The plant materials used in this study were obtained from the wild and permission was obtained to collect samples. The collection of plant materials also complied with institutional, national, or international guidelines. A total of 258 individual plants was sampled from eleven natural populations of *P. decomposita* across almost the complete regional distribution of China in 2017 prior to the flowering season. Ten–40 individual plants that were at least 10 m apart were sampled from each population. Details of the sampling are listed in Table 7 and Fig. 6. Fresh, tender, and healthy leaves were individually sampled in the wild, then immediately placed in plastic. The bags were sealed and dried using chromotropic silica gel and stored at − 20 °C until isolation of the DNA. The formal identification of the samples used in this study was performed by Shi-Quan Wang. Voucher specimens were deposited in the herbarium of Hainan Normal University.

**Table 7:** The sampling information of 11 populations of *Paeonia decomposita*

Population	Population ID	Geographical coordinate		Altitude (m)	Sample size
		Latitude(°N)	Longitude(°E)		
Danba 1	DB1	30.96503486°N	101.8592443°E	2819	21
Danba 2	DB2	30.84260025°N	101.9128609°E	2490	28
Jinchuan 1	JC1	31.48171608°N	102.0683160°E	2212	13
Jinchuan 2	JC2	31.31824367°N	102.0190106°E	2135	24
Jinchuan 3	JC3	31.28406350°N	102.0008489°E	2865	20
Jinchuan 4	JC4	31.36665014°N	101.9824393°E	2240	22
Jinchuan 5	JC5	31.46720113°N	102.1038017°E	2332	18
Maerkang 1	M1	31.99887580°N	102.0183703°E	2498	40
Maerkang 2	M2	31.88084028°N	102.2571444°E	2690	40
Maerkang 3	M3	31.92747578°N	102.1097246°E	2566	22
Maerkang 4	M4	31.91673120°N	102.1856441°E	2647	10

### DNA extraction and PCR amplification

Total genomic DNA was extracted from the leaves of each sample using the Plant Genomic DNA Kit (Tiangen Biotech, Beijing, China) in accordance with the manufacturer’s instructions. DNA concentration and quality were measured using spectrophotometry and gel electrophoresis in 1% agarose, respectively. The extracted DNA was diluted to a working concentration of 50 ng/μl then stored at − 20 °C until required. Primers previously documented and developed for tree peonies were selected for screening. These primers were screened on 12 representative samples and, after initial screening, 16 polymorphic microsatellite primer pairs [35–43] (from genomic, Table 1) producing a high degree of polymorphism and high level of amplification were selected for subsequent analysis.

SSR-PCR amplification reactions were conducted using a total volume of 10 μl consisting of 5 μl of 2 × Taq PCR MasterMix (0.1 U/μl Taq DNA Polymerase, 0.5 mM each of dNTPs, 20 mM Tris-HCl, 100 mM KCl, 3 mM MgCl_2_), 1 μl of genomic DNA template (50 ng/μl) from each accession, 3 μl of ddH_2_O, and 1 μl of each primer [labeled with a 5′ fluorescent tag (TGTAAAACGACGGCCAGT, FAM, HEX, TAMRA)]. PCR amplification was conducted using a Bio-Rad thermal cycler (Applied Biosystems) with either of two different cycling protocols, as follows: 1. Pre-denaturation at 95 °C for 5 min, followed by 10 cycles of denaturation at 95 °C for 30 s, annealing at 62–52 °C for 30 s (1 °C drop for each cycle), and extension at 72 °C for 30 s. 2. 25 cycles of denaturation at 95 °C for 30 s, annealing at 52 °C for 30 s, then extension at 72 °C for 30 s, followed by a final extension at 72 °C for 20 min. All PCR products were genotyped using capillary electrophoresis on an ABI 3730XL DNA Analyzer. The alleles of all loci were scored relative to LIZ 500, an internal product size standard, with the aid of GeneMarker Version 4.0 (Softgenetics, USA).

### Data analyses

The polymorphic information content (*PIC*) was computed using Cervus 3.0 software [73], with allelic richness (*A*_r_) and intra-population inbreeding coefficients (*F*_is_) calculated using FSTAT 2.9.3.2 software [74]. The percentage of polymorphic loci (*PPL*), number of private alleles (*N*_P_), number of observed alleles (*N*_a_), number of effective alleles (*N*_e_), Shannon’s information index (*I*), observed heterozygosity (*H*_o_), expected heterozygosity (*H*_e_), fixation index (*F*), genetic differentiation coefficient (*F*_st_), gene flow (*N*_m_), Nei’s genetic distance (NGD), genetic identity (NGI), *F*-statistics, Hardy-Weinberg equilibrium (HWE), Mantel test, Analysis of molecular variance (AMOVA), and Principal coordinate analysis (PCoA) were computed with GenAlEx 6.5 software [75]. The population genetic structure was analysed using a Bayesian clustering analysis method conducted in Structure 2.3.4 software [76]. A total of ten independent runs (*K* = 2–10) was performed with a run length of 1 × 10^5^ Markov Chain Monte Carlo (MCMC) replicates after a burn-in period of 1 × 10^5^ iterations in an admixture model with correlated allele frequency. The Δ*K* method [77] was employed to select the most appropriate *K* value and the optimal number of genetic clusters on Structure Harvester V6.0 software [78]. An unweighted pair group method with arithmetic mean (UPGMA) dendrogram was generated from cluster analysis with 1000 bootstrap replications using PHYLIP V3.67 software [79] on the basis of Nei’s genetic distance, which was then used to assess the genetic relationships among populations.

Monmonier’s maximum difference algorithm was used with BARRIER version 2.2 [80] to explore the geographical sites exhibiting maximal genetic discontinuities among populations. Sampling sits were mapped based upon geographical coordinates using this program, with barriers being indicated on the map via assessing maximum values within a population-pairwise genetic distance matrix.

## Supplementary information

**Additional file 1: Fig. S1.** The distribution of ΔK over K = 1–10.

**Additional file 2: Table S1.** Summary of Private Alleles by Population.

## Data Availability

The sequencing data of the 16 polymorphic microsatellite primers were listed in the manuscript, and no other DNA sequences were applied to this study.

## Abbreviations

AFLP: Amplified fragment length polymorphism
AMOVA: Analysis of molecular variance
ISSR: Inter simple sequence repeat
PcoA: Principal coordinate analysis
RAPD: Random amplified polymorphic DNA
RFLP: Restriction fragment length polymorphism
SRAP: Sequence-related amplified polymorphism
SSR: Simple sequence repeat
