# Supplementary figures and images for: Genetic diversity and population structure of the endangered species Paeonia decomposita endemic to China and implications for its conservation
Source: BMC Plant Biol. 2020 Nov 9;20:510. doi: 10.1186/s12870-020-02682-z (PMC7650209; doi:10.1186/s12870-020-02682-z)

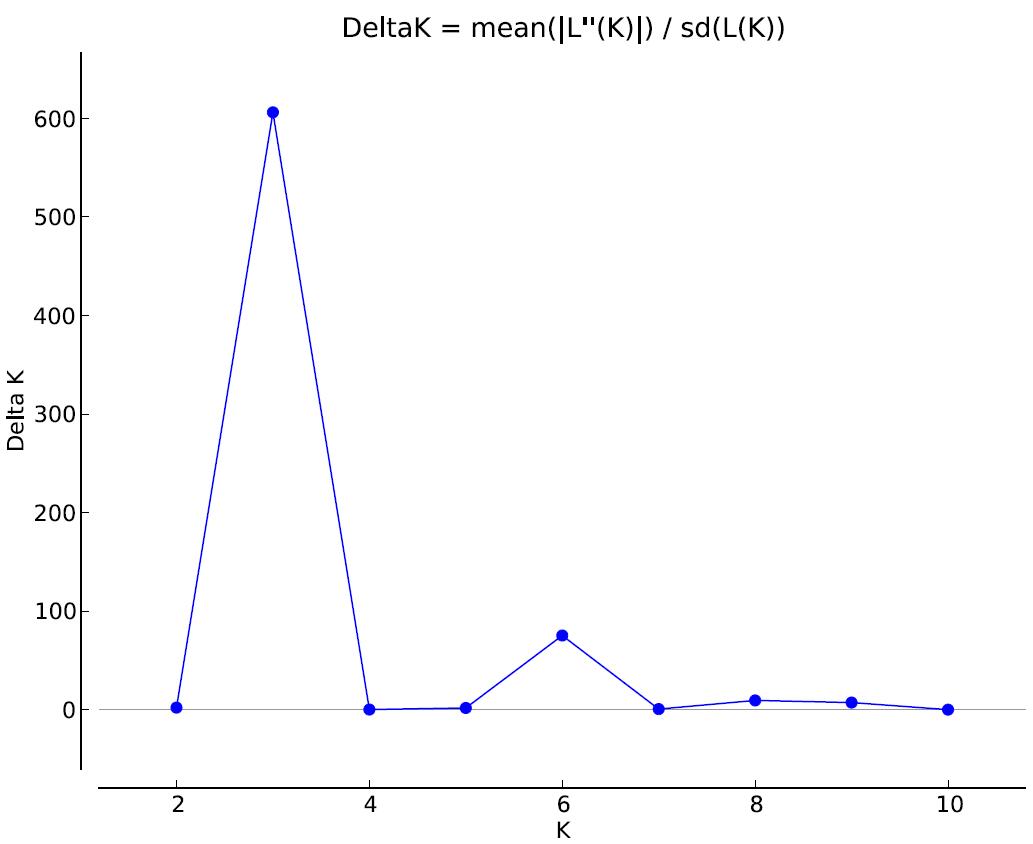


Fig.S1

Supplement: Supplementary file 1 — Additional file 1: Fig. S1. The distribution of ΔK over K = 1–10. [file 12870_2020_2682_MOESM1_ESM.docx]
