# Supplementary material for: Genetic diversity and population structure of the endangered species Paeonia decomposita endemic to China and implications for its conservation
Source: BMC Plant Biol. 2020 Nov 9;20:510. doi: 10.1186/s12870-020-02682-z (PMC7650209; doi:10.1186/s12870-020-02682-z)
Supplement: Supplementary file 2 — Additional file 2: Table S1. Summary of Private Alleles by Population. [file 12870_2020_2682_MOESM2_ESM.docx]

Table S1 Summary of Private Alleles by Population

| Number | Population | Locus | Allele | Freqency |
| --- | --- | --- | --- | --- |
| 2 | DB1 | 56A | 411 | 0.167 |
|  | DB1 | P12 | 331 | 0.050 |
| 7 | DB2 | 56A | 381 | 0.111 |
|  | DB2 | 56A | 405 | 0.019 |
|  | DB2 | PO5 | 310 | 0.018 |
|  | DB2 | P10 | 352 | 0.140 |
|  | DB2 | P10 | 354 | 0.020 |
|  | DB2 | PAG1 | 198 | 0.036 |
|  | DB2 | Pae100 | 225 | 0.019 |
| 4 | JC1 | 56A | 403 | 0.038 |
|  | JC1 | P12 | 351 | 0.042 |
|  | JC1 | P12 | 359 | 0.042 |
|  | JC1 | PS026 | 214 | 0.038 |
| 1 | JC2 | 91A | 378 | 0.045 |
| 3 | JC3 | WD09 | 214 | 0.125 |
|  | JC3 | WD09 | 220 | 0.175 |
|  | JC3 | PS004 | 318 | 0.025 |
| 1 | JC4 | PO5 | 324 | 0.205 |
| 1 | JC5 | PS004 | 316 | 0.167 |
| 1 | M1 | PS026 | 198 | 0.013 |
| 1 | M3 | 56A | 379 | 0.023 |
